# How Did the Covid-19 Pandemic Impact Cancer Prevention and Treatment? Examining Nationally Representative Survey Data

**DOI:** 10.1200/GO.22.24000

**Published:** 2022-05-05

**Authors:** Jason Semprini

**Affiliations:** ^1^ University of Iowa, Iowa City, IA

## Abstract

In March 2020, policymakers, healthcare providers, and public health professionals shifted their resources toward covid-19 prevention and mitigation. This reallocation came at the expense of other essential healthcare services, including cancer prevention and treatment. Delaying cancer services will increase the burden and cost of cancer treatment in the coming years, but the extent of these delays is unknown. As the country continues to move forward, a better understanding of how cancer services were impacted will help policymakers prioritize strategies to mitigate the adverse effects of delayed cancer prevention and treatment.

**METHODS:**

This study is among the first to use the newly released 2020 Behavioral Risk Factor Surveillance System (BRFSS) questionnaire. BRFSS is a phone-based survey and is representative of the U.S population. The BRFSS questionnaire asks about cancer screening, vaccination, and treatment services within the past year. I compared the total weighted responses in Q1, 2020 with the total weighted responses in Q4, 2020. With the latter group being exposed to six months of the covid-19 pandemic, these results serve as a baseline or “floor” estimate of how much cancer prevention and detection service patterns changed in the United States.

**RESULTS:**

Mammographies declined 4.7% (Q1 = 21,407,433 *v* Q4 = 20,403,265), pap smears declined 1.3% (Q1 = 19,464,724 *v* Q4 = 19,211,238), colorectal cancer screenings declined 9.9% (Q1 = 8,534,587 *v* Q4 = 7,693,484). While there was no estimated change in adults diagnosed with cancer, the number of adults currently receiving cancer treatment declined by 5.3% (Q1 = 485,425 *v* Q4 = 459,620).

**CONCLUSION:**

The covid-19 pandemic forced Americans to delay critical cancer prevention and treatment services. Fewer breast cancer, cervical, colorectal cancer screenings in 2020 will undoubtedly lead to more cancer diagnoses in the coming years, likely at more advanced and aggressive stages. Meanwhile cancer patients seemed to have delayed cancer treatment, but such delays cannot be sustained. Policymakers can mitigate the potential strain on cancer control systems by advancing cancer screening initiatives.

